# Multi-Floor Indoor Localization Based on Multi-Modal Sensors

**DOI:** 10.3390/s22114162

**Published:** 2022-05-30

**Authors:** Guangbing Zhou, Shugong Xu, Shunqing Zhang, Yu Wang, Chenlu Xiang

**Affiliations:** 1 School of Information and Communication Engineering, Shanghai University, Shanghai 200444, China; zhou020@shu.edu.cn (G.Z.); shunqing@shu.edu.cn (S.Z.); wangyu042@shu.edu.cn (Y.W.); xcl@shu.edu.cn (C.X.); 2 Institute of Intelligent Manufacturing, Guangdong Academy of Sciences, Guangzhou 510070, China; 3 South China Robotics Innovation Research Institute, Foshan 528300, China

**Keywords:** indoor localization, visual-based localization, WiFi signal, multi-floor, lidar SLAM

## Abstract

High-precision indoor localization is growing extremely quickly, especially for multi-floor scenarios. The data on existing indoor positioning schemes, mainly, come from wireless, visual, or lidar means, which are limited to a single sensor. With the massive deployment of WiFi access points and low-cost cameras, it is possible to combine the above three methods to achieve more accurate, complete, and reliable location results. However, the existing SLAM rapidly advances, so hybrid visual and wireless approaches take advantage of this, in a straightforward manner, without exploring their interactions. In this paper, a high-precision multi-floor indoor positioning method, based on vision, wireless signal characteristics, and lidar is proposed. In the joint scheme, we, first, use the positioning data output in lidar SLAM as the theoretical reference position for visual images; then, use a WiFi signal to estimate the rough area, with likelihood probability; and, finally, use the visual image to fine-tune the floor-estimation and location results. Based on the numerical results, we show that the proposed joint localization scheme can achieve 0.62 m of 3D localization accuracy, on average, and a 1.24-m MSE for two-dimensional tracking trajectories, with an estimation accuracy for the floor equal to 89.22%. Meanwhile, the localization process takes less than 0.25 s, which is of great importance for practical implementation.

## 1. Introduction

Location-based services (LBS) [1] have been identified as a promising technology, with which to merge social daily lives with geographic information, which triggers a paradigm shift in shopping, entertainment, and other business activities. Typical localization applications, including express delivery, turn-by-turn navigation, and travel arrangement, have, dramatically, changed people’s lives in outdoor environments, in recent years. In terms of contributing to social civilization, the improved Tiny-Yolov3 model [2] can help visually impaired people to navigate indoors and outdoors. Visually impaired people wearing Google Glass can be guided to target locations inside the building, using its floor plan [3]. A more promising area for the future lies in the indoor scenario, where indoor localization and navigation, using smartphones or Internet of things devices, have been considered important applications for this rapidly growing market. As reported in [4], the global LBS market is expected to reach a total of USD 226 billion by 2022, where 18% of the market share belongs to indoor LBS applications, with a more than 20% compound annual growth rate (CAGR).

The Global Navigation Satellite System (GNSS) can provide precise positioning services, for moving objects on the ground. Xu L. et al. [5] used GNSS to locate vehicles that were moving fast on expressways, achieving a positioning accuracy of 4–6 m. With the aid of Inertial Navigation Systems (INS), GNSS localization accuracy can be provided [6]. In the indoor scenario, Bluetooth low-energy (BLE) [7], Ultra-Wide Band (UWB) [8], 3rd-Generation Partnership Project (3GPP) long-term evolution (LTE)/5G, and WiFi signals [9,10] are adopted, instead, since the GNSS signals suffer from building shielding. Indoor-localization technology, based on static cameras, mobile cameras, wireless, IMU, and other sensor components has received increasing attention from scholars. As cameras become more affordable, as well as their integration with smart devices, visual indoor positioning is becoming popular. It is, increasingly, widely applicable in the fields of auxiliary equipment, autonomous robots, monitoring, and positioning [11]. However, reaching the same level of localization accuracy is quite challenging, due to the complexity and variability of the indoor environment, so the large-scale application of indoor-location solutions has, yet, to be realized.

With the massive deployment of low-cost digital cameras in smart entities, a potential approach to further improve the localization accuracy is to incorporate visual information, which is often referred to as visual-based localization (VBL) [12]. Simultaneous Localization and Mapping (SLAM) achieves simultaneous positioning and map construction, based on self-perception [13].

SLAM is a self-localization technology in intelligent mobile devices. It realizes its own localization and environment mapping, through a lidar-sensing environment. The lidar SLAM localization scheme is a mature theory and technology, and [14] has carried out a complete mathematical deduction and verification of the SLAM localization scheme, from the perspective of probability theory. The commonly used 2D lidar SLAM methods are GMapping and Cartographer. Gmapping adds lidar data to the proposed distribution on the basis of particle filtering positioning and puts forward the effective particle number, as the resampling constraint [15]. Cartographer is Google’s open-source indoor location technology for mobile devices, with a front end for scanning and matching as well as a back end for closed-loop detection and optimization [16]. These SLAM localization schemes can be used as a theoretical reference for wireless and visual localization schemes because they can achieve single-floor millimeter-level indoor localization.

Since the signal feature-based localization (SFBL) and the VBL schemes share similar design philosophy, e.g., to establish databases offline and perform pattern matching online, a natural extension is to, jointly, minimize the localization errors, via combining databases and matching algorithms in a brute-force manner, and the localization performance can be improved to the meter level [17] or sub-meter level, with the help of high-cost lidar. A smarter approach is to decouple SFBL and VBL processing, in a hierarchical way [18], e.g., to perform coarse-grained localization using SFBL and fine-tune the intermediate positions using VBL, and the resultant errors can be reduced to less than 2 m. This approach requires significant manpower for image-database generation and online-processing complexity. To make this more practical, ref. [19] proposes projecting the query images into a two-dimensional floor plan for pattern-matching, and [20] utilizes the special ‘EXIT’ signs, to reduce the processing complexities.

The above hybrid SFBL and VBL approaches provide a promising direction for high-precision indoor localization, by utilizing the corresponding advantages, in a separate manner. However, it fails to explore the interactions between the two schemes, especially when the localization task is mixed with multi-floor scenarios. In this paper, we propose a joint visual and wireless signal feature-based solution for high-precision multi-floor indoor localization. As shown later, our proposed scheme can utilize the coarse localization results from SFBL, to assist the later VBL procedures, which, eventually, simultaneously reduces the localization errors as well as the processing complexities. The main contributions are listed below.

- **Signal-Feature-Assisted VBL.** The conventional hybrid SFBL and VBL scheme, simply, selects some candidate regions using SFBL, to restrict the processing complexities in the VBL stage. If we regard the region index identification as a ‘hard decision’, a more reasonable scheme is to consider a ‘soft decision’, instead. Hence, in this paper, we propose a joint visual and wireless signal feature-based localization (JVWL), by considering the likelihood distribution of potential positions, which eventually helps to improve the localization accuracy.
- **Single-Floor to Multi-Floor Extension.** Different from the single-floor cases, where the horizontal dimension is neglected, multi-floor localization raises many challenging issues, regarding the existing localization mechanisms. For example, the floor structure is more or less the same for different floors, which is generally difficult for VBL. Therefore, we utilize a multi-domain translation architecture, on top of signal-feature-based coarse localization, to learn the minor changes in different floor environments.
- **Low-Complexity Few-Shot Learning.** In addition, we study a low-complexity dataset construction mechanism and, numerically, analyze the relationship between the localization accuracy and the number of sampling images. Through some numerical results, we show that high-accuracy localization results can be achieved with low-complexity few-shot learning methods.

The rest of the paper is organized, as follows. Section 2 analyzes the related work in learning-based visual localization, few-shot learning, and fusion-based localization. The entire localization system model is described in Section 3, and the proposed joint localization scheme for the multi-floor scene is discussed in Section 4. In Section 5, we present our experimental results, and the concluding remarks are provided in Section 6.

## 2. Related Works

### 2.1. Learning-Based Visual Localization

Learning-based visual localization systems perform as image regression, with a large number of datasets. For example, PoseNet has been proposed in [21], which was recognized as the first successful end-to-end pre-trained deep CNNs approach, for 6-DoF pose regression. Long-Short-Term Memory (LSTM) units have been discussed in [22], to avoid overfitting issues in the traditional CNNs approaches. Moreover, the feature-fusion mechanism was later incorporated in NetVLAD [23], with multiple-CNN-based feature extraction.

One of the most critical issues for CNN-based approaches is the limited generalization capability, e.g., the related algorithms can, hardly, adapt to the changing environment [24]. To address this, generative adversarial networks (GANs) [25] have been proposed in the conventional computer-vision tasks, including CycleGAN [26] and ComboGAN [27], which are shown to achieve superior feature-extraction abilities, compared to conventional CNNs. Motivated by this fact, ToDayGAN was proposed in [28], to use GAN-based architecture for localization, which is shown to achieve a five-meter accuracy, with a 52.9% probability, for both daytime and nighttime. Based on the ComboGAN architecture, a novel domain-invariant-feature-learning approach has been proposed in [29], and the resultant probability can be improved to 87.2%. This is, partially, because ComboGAN’s flexible combination of encoder–decoder pairs can, effectively, learn and extract domain-invariant features across multiple image domains.

Although the learning-based visual localization can outperform the conventional feature-extraction-based scheme, such as speeded-up robust features (SURF), it usually requires a huge amount of high-quality training data, e.g., hundreds of thousands of images taken from different positions, under diverse conditions, to guarantee robustness, as mentioned in [28]. To reduce the complexities in collecting high-quality images, an unpaired image-to-image translation scheme was, also, proposed in [27].

### 2.2. Few-Shot Learning

Few-shot learning is another promising approach, to reduce the requirement for high-quality images. Depending on the number of sampling images, few-shot learning [30] can, roughly, be categorized into two types, including meta-learning-based methods and transfer-learning-based methods.

Meta-learning-based methods, also known as learning to learn, aim to learn a generalized model that can be adapted to infer new classes, using only few-shot training samples. For instance, an optimization framework, to update hyper-parameters in neural networks, between the meta-training and meta-testing stages, has been proposed in [31], and an abstracted learning metric, to measure the similarities between training images and test images, has been exploited in [32]. All the above meta-learning-based methods require only a limited number of sampling data, with the episodic training strategy.

Transfer-learning-based methods [33], however, apply conventional approaches to pre-train a generalized model from the basic dataset, and are adapted to some specific tasks, with few-shot training samples. Since the classifier weights in the neural networks are critical to the adaptation, the existing transfer-learning-based mechanisms focus on analyzing the feature embeddings of few-shot samples. As an example, the mean vectors of feature embeddings have been utilized in [34], and a generalized mapping function from the feature embeddings to the weights of classifiers, has been studied in [35]. Meanwhile, an attention module, to dynamically predict the weights of classifiers, has been proposed in [36], which outperforms meta-learning-based methods, as illustrated in [37].

### 2.3. Fusion-Based Localization

As mentioned before, both the SFBL methods and the VBL schemes have pros and cons, which have not been widely implemented to date [38]. In order to be more cost-efficient, a hybrid SFBL and VBL scheme has been proposed in [39], where context information from visual data and signal features from WiFi data are fused together, to provide highly accurate localization results. A deep-fusion mechanism for wireless signals and visual images is proposed in [40], which incorporates the wavelet-transformed-signal features and a scale-invariant feature, from sampled images. The famous LASSO algorithm is adopted, to achieve 0.83-m localization accuracy. In [41], the extended naive Bayes and SURF algorithms are utilized, to extract the features of WiFi signals and visual images, respectively. A particle-filter-based fusion scheme is, then, proposed for localization estimation, which achieves 1.9-m accuracy. The above particle-filter-based fusion framework has been extended to incorporate INS signals in [17], where improved two-dimensional convolutions are applied, to generate RGB-WM image features. According to the experimental results, this fusion-based localization scheme can achieve less than 1.23-m accuracy.

The aforementioned fusion-based localization schemes can achieve superior localization accuracy, in general. However, the computational and storage costs of image processing are much more significant than the SFBL. In addition, if we consider the feature extraction and fusion complexities, the real-time fusion-based localization scheme cannot be installed on mobile devices.

### 2.4. Lidar-Slam Localization

**Figure 1:**
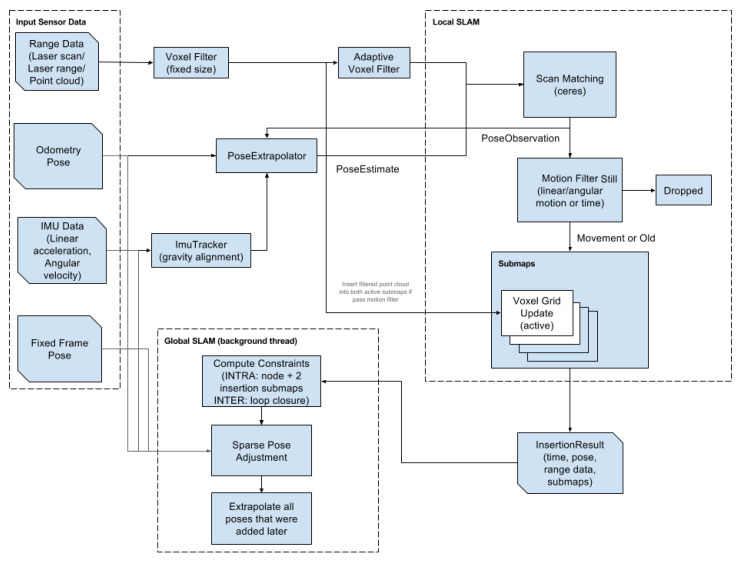
Block diagram of Cartographer scheme.

As shown in Figure 1, Cartographer is a scan-matching algorithm based on graph optimization. Cartographer incorporates laser, odometer, IMU and other multi-sensor data. Local SLAM (Frontend): laser data through voxel filtering, odometer and IMU data through track calculation. The Scan data are matched with the latest Submap, so the Scan data of this frame are inserted into the optimal position on the Submap. The Submap is updated, as new data frames are inserted. A certain amount of Scan data forms a Submap. If no new Scan is inserted into the Submap, the Submap is considered to have been created, and the next Submap is created, according to the step size. Global SLAM (Backend): each Submap has cumulative errors. The cumulative error is optimized by loop detection. If the current Scan and each created Submap is close enough, loopback detection is performed. To reduce the computation, the branch and bound method is used to search [42].

## 3. System Model

In this section, we introduce the overall procedures of the proposed JVWL scheme and discuss the construction of databases in what follows.

### 3.1. Overall Description

**Figure 2:**
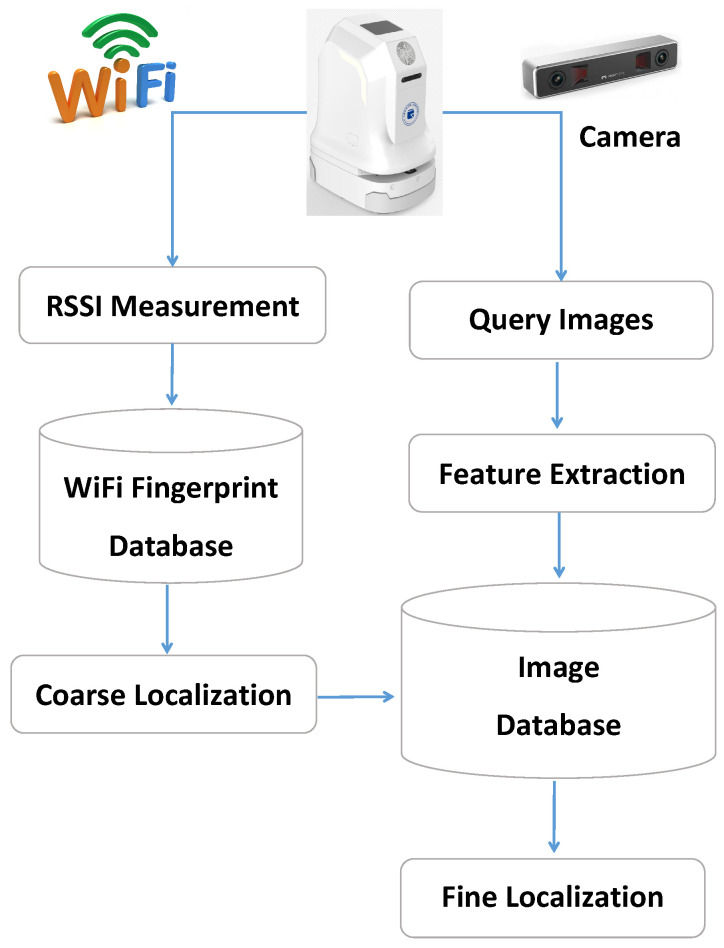
The overall procedures of the proposed JVWL scheme. It contains two stages, including WiFi RSSI-based coarse localization and visual-image-based fine localization.

As shown in Figure 2, the proposed JVWL scheme first collects $N_{s}$ sample received signal strength indications (RSSIs) from $N_{AP}$ WiFi access points, and $N_{p}$ query images with $N_{w}\times N_{l}$ pixels and $N_{RGB}$ color channels from on-device cameras, where the corresponding observations are denoted as $R\left(L\right)\in R^{N_{s}\times N_{\mathrm{AP}}}$ and $I\left(L\right)\in Z^{N_{p}\times N_{w}\times N_{l}\times N_{RGB}}$ for a given location L, respectively. RSSIs of $N_{RP}$ reference points (RPs) are collected offline to construct WiFi fingerprint database, ${\mathrm{DB}}_{W}$, which consists of $N_{RP}$ RP locations, $\left\{L_{RP}^{i}\right\}$, and the measured RSSIs, $\left\{R\left(L_{RP}^{i}\right)\right\}$. The image database, ${\mathrm{DB}}_{I}$, is constructed in a similar manner, which contains the location $L_{I}$, and the associated images, $\left\{I\left(L_{I}\right)\right\}$.

The entire localization procedures consist of a WiFi RSSI-based coarse localization and a visual-image-based fine localization (kindly note that the above RSSI and visual information can easily be obtained by the sensors on the mobile robots, such as WiFi receivers and cameras.), as explained below.

**Figure 3:**
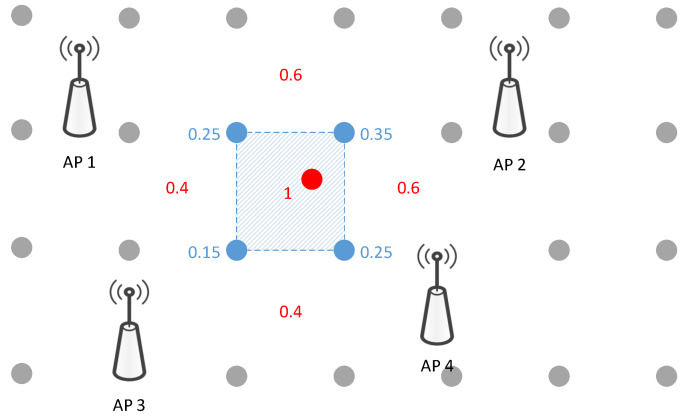
The illustration of coarse localization. The nearest $N_{p}$ RPs are selected by the KNN algorithm and form a circle area, which is the coarse localization area. The gray dots and red dots are RP positions and test positions, respectively.

- *Coarse Localization f(·)* In the coarse localization, the proposed JVWL scheme first computes the likelihood probability with respect to (w.r.t.) $N_{RP}$ RPs, i.e., ${\hat{p}}_{RP}\left(L\right)=\left[{\hat{p}}_{RP}^{1}\left(L\right),\ldots ,{\hat{p}}_{RP}^{i}\left(L\right),\ldots ,{\hat{p}}_{RP}^{N_{RP}}\left(L\right)\right]$, by inquiring the WiFi fingerprint database, ${\mathrm{DB}}_{W}$. By comparison with the observed WiFi RSSIs, the likelihood probability w.r.t. the ith RP can be obtained via a standard support vector machine (SVM) scheme, which has proven to be effective in fingerprint classification tasks [43], e.g.,
  (1) $$\begin{array}{c} {\hat{p}}_{RP}^{i}\left(L\right)=f_{1}\left(R\left(L\right),{\mathrm{DB}}_{W}\right). \end{array}$$
  To reduce the searching complexity of the latter fine localization, we partition the target areas into $N_{A}$ consecutive areas based on $N_{RP}$ RPs, $\left\{A^{j}\left(\{L_{RP}^{i}\}\right)\right\}$, as shown in Figure 3. The likelihood probabilities of $N_{A}$ areas are, as follows:
  (2) $$\begin{array}{c} {\hat{p}}_{A}\left(L\right)=\left[{\hat{p}}_{A}^{1}\left(L\right),\ldots ,{\hat{p}}_{A}^{j}\left(L\right),\ldots ,{\hat{p}}_{A}^{N_{A}}\left(L\right)\right] \end{array}$$
  This can be calculated by summing over the likelihood probabilities of RPs, where each element ${\hat{p}}_{A}^{j}\left(L\right)$ is given by,
  (3) $$\begin{array}{c} {\hat{p}}_{A}^{j}\left(L\right)=f_{2}\left({\hat{p}}_{RP}\left(L\right)\right)=\sum_{i\in A^{j}\left(\{L_{RP}^{i}\}\right)}{\hat{p}}_{RP}^{i}\left(L\right). \end{array}$$
  The coarse localization results are, thus, given by selecting $J^{⋆}$ most possible areas according to the likelihood probabilities, ${\hat{p}}_{A}\left(L\right)$. Mathematically, if we denote $\Omega_{j}^{⋆}\left(L\right)$ and $\bar{\Omega_{j}^{⋆}\left(L\right)}$ to be the index set of selected areas and its complementary set, the candidate localization area $A^{⋆}\left(L\right)$ and the corresponding likelihood probability ${\hat{p}}_{A^{⋆}}\left(L\right)$ can be expressed as
  (4) $$\begin{array}{ccc} A^{⋆}\left(L\right) & = & \underset{j\in \Omega_{j}^{⋆}\left(L\right)}{⋃}A^{j}\left(\{L_{RP}^{i}\}\right), \end{array}$$
  (5) $$\begin{array}{ccc} {\hat{p}}_{A^{⋆}}\left(L\right) & = & \left\{{\hat{p}}_{A}^{j}\left(L\right)\right\},\;\forall j\in \Omega_{j}^{⋆}, \end{array}$$
  where ${\hat{p}}_{A}^{j}\left(L\right)\geq {\hat{p}}_{A}^{j^{\prime }}\left(L\right)$ for any $j\in \Omega_{j}^{⋆}\left(L\right)$ and $j^{\prime }\in \bar{\Omega_{j}^{⋆}\left(L\right)}$, and the cardinality of $\Omega_{j}^{⋆}\left(L\right)$ is $J^{⋆}$. By cascading (1)–(5), we denote the entire coarse localization process as
  $$\begin{array}{c} \left(A^{⋆}\left(L\right),{\hat{p}}_{A^{⋆}}\left(L\right)\right)=f\left(R\left(L\right),{\mathrm{DB}}_{W},\left\{A^{j}\left(\{L_{RP}^{i}\}\right)\right\}\right). \end{array}$$
- *Fine Localization g(·)* In the fine localization, the proposed JVWL scheme maps the $N_{p}$ query images, I(L), to the estimated location, $\hat{L}\in A^{⋆}\left(L\right)$, according to the image database ${\mathrm{DB}}_{I}$. To control the searching complexity, we only use a subset of the entire image database in the practical deployment, e.g.,
  (6) $$\begin{array}{c} {\mathrm{DB}}_{I}\left(A^{⋆}\left(L\right)\right)≜\left\{I\left(L_{I}\right),\forall L_{I}\in A^{⋆}\left(L\right)\right\}\subset {\mathrm{DB}}_{I}, \end{array}$$
  and the mathematical expression of the fine localization process is given by
  (7) $$\begin{array}{c} \hat{L}=g\left(I\left(L\right),{\mathrm{DB}}_{I}\left(A^{⋆}\left(L\right)\right)\right). \end{array}$$

### 3.2. Database Construction

In order to construct the databases ${\mathrm{DB}}_{W}$ and ${\mathrm{DB}}_{I}$, a site survey of WiFi RSSI fingerprints and camera images is conducted with $N_{RP}$ and $|L_{I}|$ positions, respectively. To make a more reliable database, $N_{W}$ rounds of RSSIs collections and $N_{IR}$ rounds of images are performed to construct ${\mathrm{DB}}_{W}$ and ${\mathrm{DB}}_{I}$, e.g., ${\mathrm{DB}}_{W}=\left\{\left(R^{N_{W}}\left(L_{RP}^{i}\right),L_{RP}^{i}\right)\right\}$ and ${\mathrm{DB}}_{I}=\left\{\left(I^{N_{I}}\left(L_{I}\right),L_{I}\right)\right\}$, respectively. Since ${\mathrm{DB}}_{I}\left(A^{⋆}\left(L\right)\right)$ is equal to

(8) $$\begin{array}{c} \cup_{j\in \Omega_{j}^{⋆}\left(L\right)}{\mathrm{DB}}_{I}\left(A^{j}\left(\{L_{RP}^{i}\}\right)\right) \end{array}$$

we can partition the image database ${\mathrm{DB}}_{I}$ into $N_{A}$ parts in the offline stage, e.g.,

(9) $$\begin{array}{c} \left\{{\mathrm{DB}}_{I}\left(A^{j}\left(\{L_{RP}^{i}\}\right)\right)\right\} \end{array}$$

and efficiently construct ${\mathrm{DB}}_{I}\left(A^{⋆}\left(L\right)\right)$ in the online stage. For the convenience of data collection and future update, a mobile robot equipped with WiFi, camera, lidar and IMU sensors are used to construct ${\mathrm{DB}}_{W}$ and ${\mathrm{DB}}_{I}$, with corresponding ground-truth positions. More implementation details are presented in Section 5.

## 4. Proposed Multi-Floor Scheme

In this section, we describe the problem formulation and the proposed multi-floor-localization scheme. To be more specific, we propose a joint-optimization framework for visual and wireless localization, based on which a novel neural network structure and loss function are, then, presented.

### 4.1. Problem Formulation

To obtain a reliable localization error performance, we introduce the subscript *k* to the ground-truth and estimated locations and formulate the multi-floor localization problem, as follows.

**Problem** **1** (Multi-Floor Localization).

(10) $$\begin{array}{ccc} \underset{{\hat{L}}_{k}}{\mathrm{minimize}} & & \frac{1}{K}\sum_{k=1}^{K}\min_{j\in \Omega_{j}^{⋆}\left({\hat{L}}_{k}\right)}\frac{∥{\hat{L}}_{k}-L_{k}{∥}_{2}^{2}}{{\hat{p}}_{A}^{j}\left({\hat{L}}_{k}\right)}\\ \mathrm{subject}\mathrm{to} & & \left(A^{⋆}\left({\hat{L}}_{k}\right),{\hat{p}}_{A^{⋆}}\left({\hat{L}}_{k}\right)\right)=f(R\left({\hat{L}}_{k}\right), \end{array}$$

(11) $$\begin{array}{ccc} & & {\mathrm{DB}}_{W},\left\{A^{j}\left(\{L_{RP}^{i}\}\right)\right\}), \end{array}$$

(12) $$\begin{array}{ccc} & & {\hat{L}}_{k},{\hat{C}}_{k}=g\left(I\left(L_{k}\right),{\mathrm{DB}}_{I}\left(A^{⋆}\left({\hat{L}}_{k}\right)\right)\right), \end{array}$$

(13) $$\begin{array}{ccc} & & {\hat{L}}_{k}\in A^{⋆}\left({\hat{L}}_{k}\right),\forall k, \end{array}$$

*where K is the total number of localization tasks, ${\hat{C}}_{k}$ represents the category of the current user’s floor.*

We have a large-scale dataset $D_{base}$ from ${\mathrm{DB}}_{I}$, containing many-shot-labeled examples from each base class (domain) $C_{base}$ and a small-scale dataset $D_{novel}$ of only few-shot-labeled examples. The task of few-shot learning in our scheme is to learn a robust classifier using the few-shot-labeled examples in $D_{novel}$, with the examples in $D_{base}$ as auxiliary data.

The main component of all few-shot algorithms is a feature extractor $g_{\theta }\left(\cdot \right)$, which is a convolutional neural network with parameters θ. Given an image x, the feature extractor will output a d-dimensional feature $g_{\theta }\left(x\right)$. How can the feature extractor $g_{\theta }\left(\cdot \right)$ learn image features that can be readily exploited for novel classes with few training data during the second stage? With this goal in mind, we propose leveraging the recent progress in GAN feature learning, to further improve the current few-shot-learning approaches.

Based on the above multi-floor-localization problem, we decompose the original problem into two stages. It computes the feature vector, with respect to database images in the first stage, and compares the feature with every feature vector in the database, to obtain the final results in the second stage. The corresponding mathematical formulation is given below.

**Problem** **2** (Regression-Based Localization).

(14) $$\begin{array}{ccc} \underset{g_{\theta }\left(\cdot \right),{\hat{L}}_{k}}{\mathrm{minimize}} & & \frac{1}{K}\sum_{k=1}^{K}\min_{j\in \Omega_{j}^{⋆}\left({\hat{L}}_{k}\right)}\frac{∥{\hat{L}}_{k}-L_{k}{∥}_{2}^{2}}{{\hat{p}}_{A}^{j}\left({\hat{L}}_{k}\right)}\\ \mathrm{subject}\mathrm{to} & & \left(A^{⋆}\left({\hat{L}}_{k}\right),{\hat{p}}_{A^{⋆}}\left({\hat{L}}_{k}\right)\right)=f(R\left({\hat{L}}_{k}\right), \end{array}$$

(15) $$\begin{array}{ccc} & & {\mathrm{DB}}_{W},\left\{A^{j}\left(\{L_{RP}^{i}\}\right)\right\}), \end{array}$$

(16) $$\begin{array}{ccc} & & v_{k}=g_{\theta }\left(I\left(L_{k}\right),{\mathrm{DB}}_{I}\left(A^{⋆}\left({\hat{L}}_{k}\right)\right)\right), \end{array}$$

(17) $$\begin{array}{ccc} & & {\hat{L}}_{k},{\hat{C}}_{k}=\tilde{g}\left(v_{k}\right), \end{array}$$

(18) $$\begin{array}{ccc} & & {\hat{L}}_{k}\in A^{⋆}\left({\hat{L}}_{k}\right),\forall k, \end{array}$$

*where $v_{k}$ denotes the extracted feature vector of all the images at the current floor.*

In the formulation of Problem 2, g(·) has been decomposed into two simplified functions, $g_{\theta }\left(\cdot \right)$ and $\tilde{g}\left(\cdot \right)$. The function $g_{\theta }\left(\cdot \right)$ and $\tilde{g}\left(\cdot \right)$ are modeled as a typical feature-extract and feature-retrieve problem, to predict the precise location and floor index. The function $g_{\theta }\left(\cdot \right)$ consists of a generator *G* and discriminator *D*, for each domain.

In the feature-extraction model $g_{\theta }\left(\cdot \right)$, assuming that a generator function $G_{AB}$ that can transform domain A into domain B exists, such that $b=G_{AB}\left(a\right)$, a∈A, b∈B. Additionally, another generator $G_{BA}$ transforms in the reverse direction, that is, $\tilde{a}=G_{BA}\left(\tilde{b}\right)$. Similarly, $G_{BA}$ should, also, transform domain B to domain A.

In the function $\tilde{g}\left(\cdot \right)$, we first use the corresponding feature extractor to extract the feature vector for the query image, then compare the feature with every feature vector in the database, using a cosine distance metric, choosing the most similar feature as the retrieval result. Compared with the conventional visual-based localization technologies, the proposed multi-floor-localization scheme only needs to evaluate over the potential area $A^{⋆}\left({\hat{L}}_{k}\right)$, which undoubtedly reduces the computational complexity.

**Problem** **3** (GAN-Based Localization).

(19) $$\begin{array}{c} \begin{array}{c} g_{\theta }{\left(\cdot \right)}^{*}=arg\;\underset{g_{\theta }\left(\cdot \right)}{min}\;\{L_{Gan}\left(G_{AB},D_{B}\right)+L_{Gan}\left(G_{BA},D_{A}\right)\\ +\lambda_{1}L_{cyc}\left(G_{AB},G_{BA}\right)+\lambda_{2}L_{fea}\left(E_{A},E_{B},G_{AB},G_{BA}\right)\} \end{array} \end{array}$$

*where $\lambda_{1}$ and $\lambda_{2}$ are hyperparameters used to balance the training direction.*

We apply a cycle consistency $L_{1}$ loss between domain *A* and *B*, as follows:

(20) $$\begin{array}{c} \begin{array}{c} L_{cyc}\left(G_{AB},G_{BA}\right)=E_{a-p_{\left(a\right)}}\left[{\|G_{BA}\left(G_{AB}\left(a\right)-a\right\|}_{1}\right]+\\ E_{b-p_{\left(b\right)}}\left[{\|G_{AB}\left(G_{BA}\left(b\right)-b\right\|}_{1}\right], \end{array} \end{array}$$

where $a-p_{\left(a\right)}$ and $b-p_{\left(b\right)}$ are the image collections at the correspond domain, and *E* is the expectation function. Discriminator $D_{A}$ and $D_{B}$ work in each domain and try to discriminate between $A,\tilde{A}$ and $B,\tilde{B}$, respectively. We apply adversarial losses, such as:

(21) $$\begin{array}{c} \begin{array}{c} L_{Gan}\left(G_{AB},D_{B}\right)=E_{b-p_{\left(b\right)}}\left[logD_{B}\left(b\right)\right]\\ +E_{a-p_{\left(a\right)}}\left[1-logD_{B}\left(G_{AB}\left(a\right)\right)\right], \end{array} \end{array}$$

(22) $$\begin{array}{c} \begin{array}{c} L_{Gan}\left(G_{BA},D_{A}\right)=E_{a-p_{\left(a\right)}}\left[logD_{A}\left(a\right)\right]\\ +E_{b-p_{\left(b\right)}}\left[1-logD_{A}\left(G_{BA}\left(b\right)\right)\right]. \end{array} \end{array}$$

To improve the training efficiency and make the model more practical for the localization task, we adopt a feature consistency loss of [29], built on the encoded features of different domains.

(23) $$\begin{array}{c} \begin{array}{c} L_{fea}\left(E_{A},E_{B},G_{AB},G_{BA}\right)=\\ E_{a-p_{\left(a\right)}}\left[{\|E_{B}\left(G_{AB}\left(a\right)\right)-E_{A}\left(a\right)\|}_{2}\right]\\ +E_{b-p_{\left(b\right)}}\left[{\|E_{A}\left(G_{BA}\left(b\right)\right)-E_{B}\left(b\right)\|}_{2}\right], \end{array} \end{array}$$

where $E_{A}$ and $E_{B}$ denote the encoder of the generator in domain *A* and domain *B*, respectively.

### 4.2. Multi-Floor-Model Architecture

**Figure 4:**
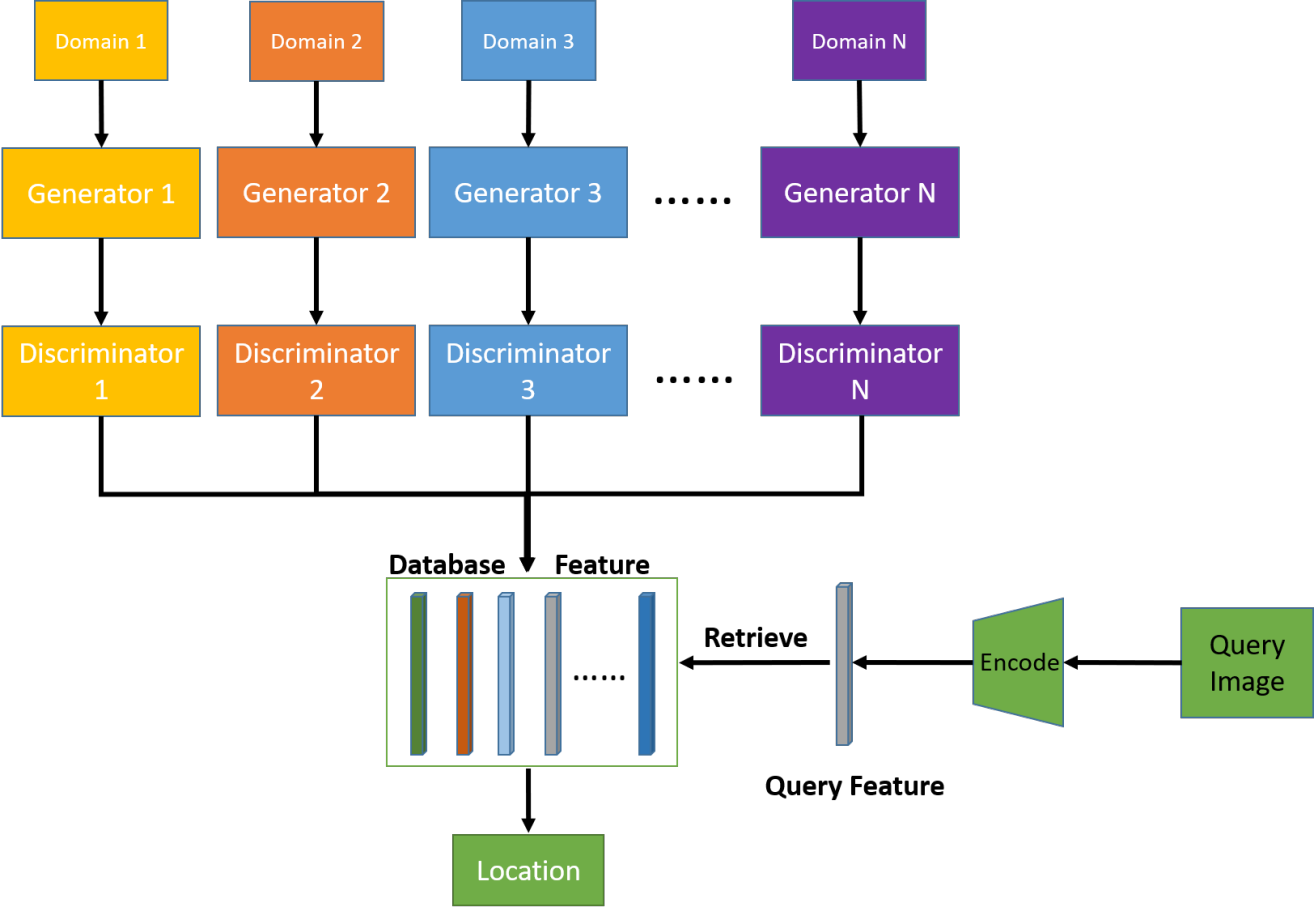
The overall procedures of the proposed multi-floor-localization scheme. The query image is encoded as a feature vector and, then, used to retrieve the feature and image with the largest similarity in the database.

The overall procedures of the proposed multi-floor-localization scheme are shown in Figure 4, including the GAN-based feature-extraction model and the retrieval-based localization model.

**Figure 5:**
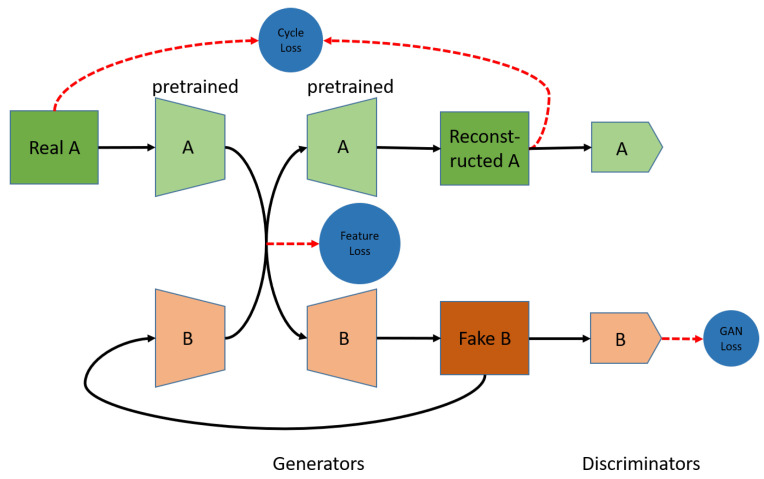
The proposed GAN-based localization architecture, which consists of generators and discriminators. The generators are divided into domain-specific pairs of encoders and decoders for each domain. The architecture of translation is A → B, where A and B are randomly selected. The pass B → A is performed in the same fashion.

In the GAN-based feature-extraction model $g_{\theta }\left(\cdot \right)$, we adopt the basic framework of multi-domain translation architecture ComboGAN [27]. Our proposed GAN-based localization architecture is shown as Figure 5, including GAN loss $L_{Gan}$, cycle consistency loss $L_{cyc}$, and feature-consistency loss $L_{fea}$. We design the loss function L of the neural network, according to the previous problem formulation, which is given by

(24) $$\begin{array}{c} L=min\;\{L_{Gan}+\lambda_{1}L_{cyc}+\lambda_{2}L_{fea}\}. \end{array}$$

The detailed configurations and parameters of our neural network are listed in Table 1 (Note that each convolutional layer in the Generators part corresponds to ‘Conv-InstanceNorm-ReLU’ )sequence. The transposed convolutional layer is denoted by Uconv1-3. The residual basic block is denoted as Res1-9.

The training procedure involves focusing on two of the total *N* domains at a time. At the beginning of each iteration, we selected two domains A,B∈{1,2,⋯,N}, A≠B from *N* domains, uniformly and at random. To make the GAN-based feature-extraction model more practical, for few-shot localization tasks, we proposed a training strategy based on a pre-trained model. We, first, trained a model using a large amount of labeled data from the base domains, encoding the knowledge from base domain data into the pre-trained model. Then, the pre-trained model was adopted as a feature extractor, to generate the feature embeddings of the labeled few-shot samples from the novel domain, which can be, directly, used as the initialization of the encoder for further fine-tuning.

Our retrieval-based localization is based on GAN-based feature learning, as shown in Figure 4, which can better describe the function g(·). In the online stage, we trained the GAN-based feature-extraction model mentioned above, to pre-encode each database image into a one-dimensional vector, which can avoid redundant calculations. In the offline stage, we, first, used the corresponding trained encoder networks to extract features for the query image, then compared the features with every feature vector in the database, using a cosine distance metric. We chose the image with the most similar features, as the retrieval result.

## 5. Experiment Results

In this section, we conduct several numerical experiments to show the effectiveness of our proposed system.

### 5.1. Experimental Environment

**Figure 6:**
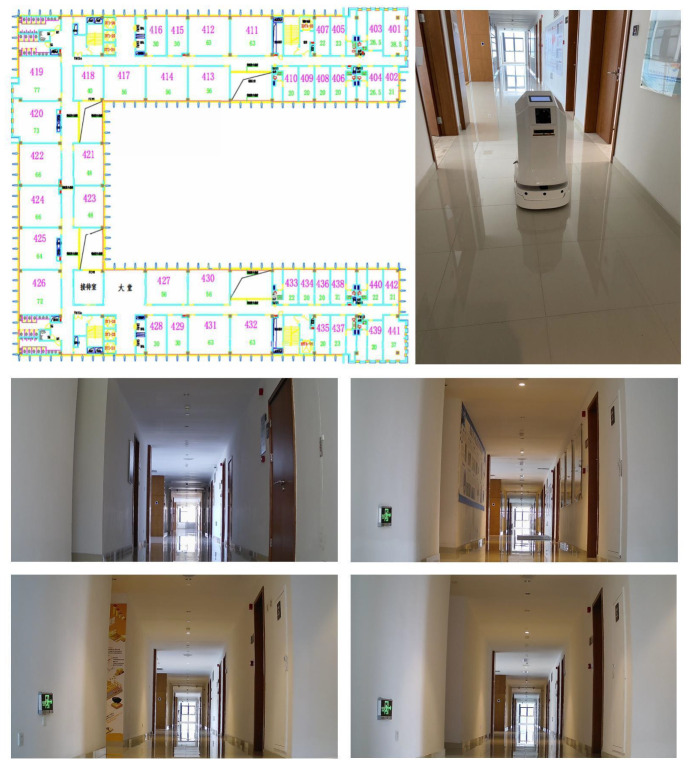
The layout of the experimental corridor environment, the mobile robot constructing ${\mathrm{DB}}_{W}$ and ${\mathrm{DB}}_{I}$, automatically, and several image samples from ${\mathrm{DB}}_{I}$.

The proposed localization scheme was verified in the corridor environment of a typical office building, as shown in Figure 6, which contains six floors, with 4000 square meters of area for each floor. The layout of each floor is more or less the same, with dramatically changing lighting conditions throughout the day. Meanwhile, the WiFi signals are, generally, unreliable, due to the regular daily activities of working staff. In the above settings, both the SFBL and the VBL schemes cannot achieve satisfying localization results, which is a great challenge for the fusion-based localization schemes.

**Figure 7:**
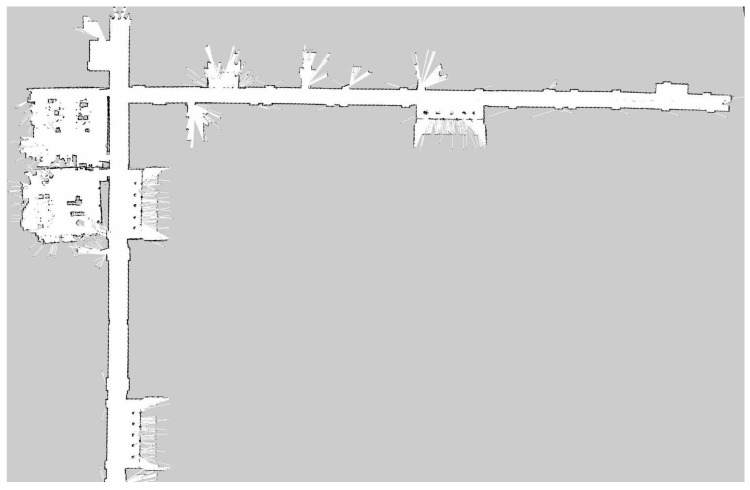
Partial SLAM map for floor 4.

To obtain the ground-truth positions and establish the databases, e.g., ${\mathrm{DB}}_{W}$ and ${\mathrm{DB}}_{I}$, we conducted the following implementation works. First, the Cartographer was used to build SLAM maps on each floor, as shown in Figure 7. The mobile robot could obtain its own positioning in real-time, and the reference coordinate origin was the starting point for mapping on each floor. Secondly, the coordinates of the camera and IMU module, relative to the robot center point, were calculated according to their installation positions. In SLAM, the transformation matrix from an image-coordinate system to a laser-coordinate system was calculated, and the image, IMU, wireless signal, and laser data are aligned. Third, several reference points were set on the 2D SLAM diagram to plan the s-shaped path of the mobile robot (the S-shaped path increases the z-axis Angle data in IMU), and the S-shaped trajectory passed through all the reference points. Finally, we set up several ramps, so that the height varied and the positioning accuracy was measured, according to the 3D distance. To ensure the localization accuracy, the mobile robot periodically collected wireless signals and image data, to keep ${\mathrm{DB}}_{W}$ and ${\mathrm{DB}}_{I}$ updated.

All sampled images in our experiments were scaled to 286×286 pixels, and randomly cropped to 256×256 pixels, according to [28], for training and testing. In the training stage, learning rates were fixed at 0.0002 for generators and 0.0001 for discriminators, for the first half, and this, linearly, decreased to zero, during the second half. The batch size was fixed to 1 and the number of epochs was chosen as 100×N. In our experiments, the data-training processes were conducted on a localization server with NVIDIA Titan X GPU and the Pytorch platform. Other detailed parameter values are listed in Table 2. Kindly note that when the offline training processes were completed, we could provide the online localization service, immediately.

### 5.2. Localization Accuracy and Computational Complexity

In the following experiments, we, first, investigated the localization accuracy, in terms of the cumulative distribution function (CDF) of 3D distance errors, to show the effectiveness of the proposed JVWL algorithm. To obtain an in-depth understanding, we plotted the estimation accuracy of floors and the corresponding mean squared errors (MSE) of estimated two-dimensional tracking trajectories (For simplicity, we only calculated the MSE of tracking trajectories, when the floor estimation was correct). Moreover, we, also, compared the associated computational complexities, in terms of the total computational times. All the above experiments were compared with the following two baseline schemes, e.g., *Baseline 1*, an SVM-based, WiFi-only localization scheme, and *Baseline 2*, a WiFi and vision-integrated scheme, as proposed in [20].

**Figure 8:**
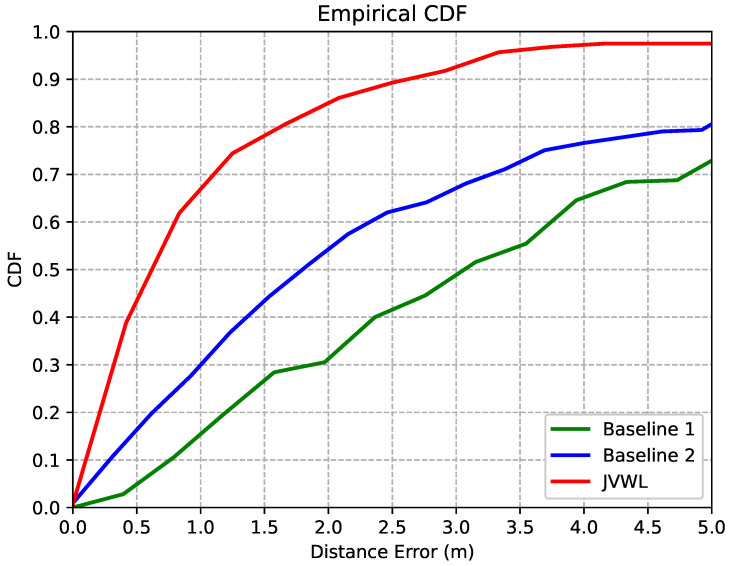
CDF of localization errors for different localization methods.

In Figure 8, we compare the localization accuracy in terms of the 3D distance errors of the proposed JVWL scheme with baselines. As shown in this figure, the proposed method (red) can achieve a 0.62-m localization accuracy with a CDF equal to 0.5, which achieves more accurate and reliable localization results than *Baseline 1* (green, 1.78 m) and *Baseline 2* (blue, 3.12 m).

**Figure 9:**
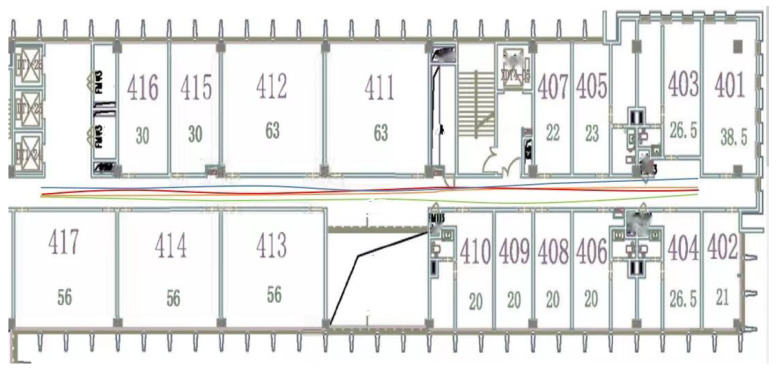
Localization trajectories for different schemes, including ground truth (yellow), JVWL (red), *Baseline 1* (green), and *Baseline 2* (blue).

In Figure 9, we plotted a snapshot view of tracking trajectories for different localization schemes, where our proposed JVML scheme (red) is closer to the ground truth (yellow) than other schemes. The detailed estimation accuracy of floors and the corresponding MSE of the estimated two-dimensional tracking trajectories are summarized in Table 3. Numerically, the proposed JVWL scheme achieves an 89.22% estimation accuracy of floors, which is far superior to *Baseline 1* (57.93%) and *Baseline 2* (66.87%). In addition, we show that the proposed JVWL scheme can achieve an MSE of estimated tracking trajectory up to 1.24 m, which outperforms *Baseline 1* (3.43 m) and *Baseline 2* (2.64 m).

Although the proposed scheme provides a satisfactory localization performance in terms of 3D distance errors, the estimation accuracy of floors, and the corresponding MSE of two-dimensional tracking trajectory, the implementation complexity is still open. In this experiment, we compared the total computational time cost with two baseline schemes. As listed in Table 3 The floor estimation accuracy of Baseline 1 is less than 60% and the MSE result is more than 3 m, which is not suitable for practical implementation., the average running time of the proposed JVWL scheme is around 0.25 s, which is five times less than *Baseline 2*. Meanwhile, the memory requirement for the proposed JVWL scheme is around 10 MB, which is much lower than *Baseline 2*. This is due to the fact that the matching algorithm adopted in *Baseline 2* requires significant storage and searching abilities.

### 5.3. Effects of Grid Sizes

**Figure 10:**
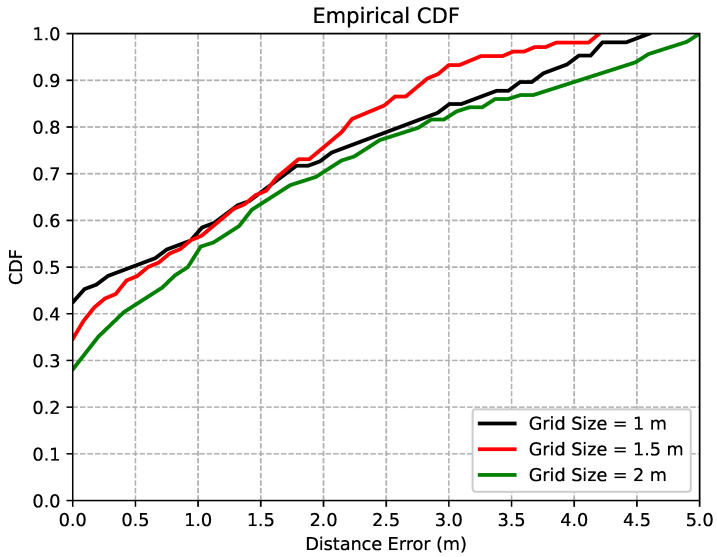
CDF of localization errors, for different training cell sizes. Three training datasets with different cell sizes were tested, to explore the most efficient deployment method.

In this experiment, we investigated the grid size effects to balance the localization accuracy and the associated deployment cost. Distances between adjacent RPs were selected as 1 m, 1.5 m, and 2 m, respectively. In Figure 10, we compared the 3D distance errors of the proposed JVWL scheme under different grid sizes. As shown in this figure, we were able to achieve 0.62 m, 0.83 m, and 1.19 m for grid sizes of 1 m, 1.5 m, and 2 m, respectively. Since the deployment cost of data collection and labeling for the grid size of 1.5 m was around 50% more than the grid size of 1 m and the localization accuracy improved by only 25.3%, we recommend choosing 1.5 m, as the grid size for the practical database construction.

### 5.4. Effects of Few-Shot

To demonstrate the effects of few-shot learning, we selected different “few-shot” approaches, e.g., to select 10%/20% of sampling images, uniformly (denoted as Uni_10 and Uni_20) and randomly (denoted as Ran_10 and Ran_20), to construct the training dataset.

**Figure 11:**
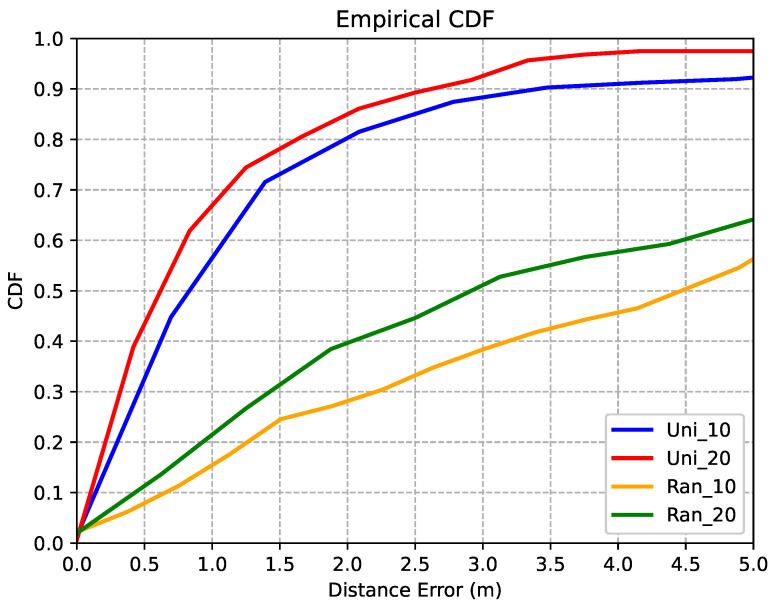
The CDF curves of 3D distance errors of different training sets for the proposed JVWL algorithm, in an experimental corridor environment.

In Figure 11 and Table 4, we compare the CDF of 3D distance errors, the estimation accuracy of floors, and the corresponding MSE of two-dimensional tracking trajectories of different “few-shot” approaches. As shown in Figure 11, selecting the sampling images uniformly is much better than selecting them randomly, and the averaged 3D distance errors can be improved, changing from more than 3 m to less than 1 m. A similar phenomenon occurs for the estimation accuracy of floors and the corresponding MSE of two-dimensional tracking trajectories, as listed in Table 4. For example, the estimation accuracy can be improved from less than 65% to more than 85%, and the corresponding MSE can be improved from more than 4.5 m to less than 2 m.

**Figure 12:**
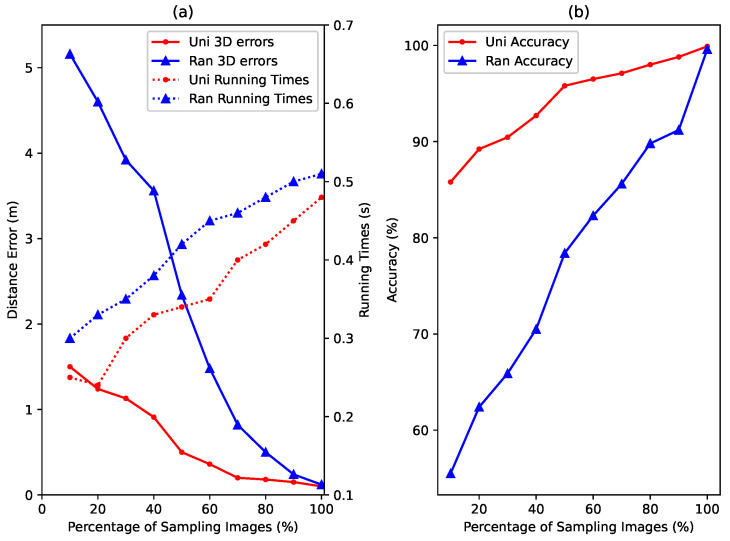
Influences of distribution of few-shot samples in our experiments. (**a**) represent results for localization distance error and average time consumption for different few-shot sample distributions. (**b**) shows results for the estimation accuracy of floors for different distributions of few-shot samples.

In Figure 12, we, linearly, increased the percentages of the sampling image selection, to demonstrate the corresponding “few-shot” effects. Intuitively, with more sampling images in the training dataset, the 3D distance errors and the estimation accuracy of floors can be improved. Moreover, as shown in Figure 12, the running times are controllable, when the percentages of sampling image selection are less than 20%. Based on the above results, we believe that our proposed JVWL scheme can, effectively, utilize few-shot samples, and 20% will be a reasonable number.

## 6. Conclusions and Discussion

In this paper, we propose a joint visual- and wireless-signal feature-based approach, for high-precision multi-floor indoor localization. By applying a hybrid coarse and fine localization framework, we could formulate the JVWL problem, accordingly. Through some theoretical analysis, a GAN-based-deep-learning scheme is proposed, for multi-floor localization architecture. Compared with the conventional SFBL and VBL schemes, our localization scheme could achieve 0.62 m 3D localization accuracy on average, and 1.24 m MSE of two-dimensional tracking trajectories, with a floor-estimation accuracy equal to 89.22%, which significantly improves the localization accuracy in the multi-floor scenarios. Meanwhile, the proposed JVWL scheme takes less than 0.25 s for the localization process, which is five times less than conventional WiFi and vision integrated schemes.

Compared with the industrial-laser SLAM solution, the JVWL solution has some advantages. First, the JVWL solution solves the multi-layer localization that laser SLAM cannot handle, especially the symmetrical environment of each floor. Secondly, JVWL scheme is suitable for people’s livelihoods, such as in service positionings, which is a low-cost and efficient positioning scheme. For example, it can be used in shopping guidance in shopping malls, personnel positioning in COVID-19 makeshift hospitals, positioning in construction site clusters, etc. Finally, the JVWL scheme is convenient for mobile terminal applications.

Our proposed localization system, still, has limitations, such as a low positioning accuracy, the need to build fingerprint database, an offline map, etc. Although this accuracy is sufficient for ordinary scenes, it is, still, difficult for industrial and special scenes. Constructing a fingerprint database is complicated and heavy work, which is a challenge for field implementation. Lidar SLAM maps can be built by remote-controlled mobile robots, or by carrying lidar in a backpack. In the future, we plan to fuse visual, wireless, and lidar data through loose coupling, hoping to make improvements in terms of cost reductions, ease of use, accuracy, and other indicators.

## Figures and Tables

**Table 1:** An Overview of Network Configuration and Parameters.

Module	Layers	Parameters
Generators	Conv1	256×256×64
	Conv2	128×128×128
	Conv3	64×64×256
	Res1-9	64×64×256
	Uconv1	64×64×256
	Uconv2	128×128×128
	Uconv3	256×256×3
Discriminator	Conv1	128×128×64
	Conv2	64×64×128
	Conv3	32×32×256
	Conv4	16×16×512
	Conv5	16×16×1

**Table 2:** The detailed parameter values of the experiments.

Parameter	Value	Parameter	Value
$N_{s}$	50	$N_{AP}$	5
$N_{p}$	492	$N_{w}$	752
$N_{l}$	780	$N_{RGB}$	3
$N_{RP}$	24	$N_{A}$	15
$N_{W}$	2	$N_{I}$	2
*J* ${}^{⋆}$	4	*N*	4
GPU	NVIDIA Titan X	Platform	Pytorch

**Table 3:** Localization accuracy, total running time, and memory space comparison for different localization methods.

Methods	Running Time	Memory Space	Accuracy	MSE
*Baseline 1*	0.01 s	4.8 MB	57.93%	3.43 m
*Baseline 2*	1.2 s	25 MB	66.87%	2.64 m
*JVWL*	0.25 s	10 MB	89.22%	1.24 m

**Table 4:** Localization accuracy with different numbers and distribution of few-shot samples.

Few-Shot Samples	Accuracy	MSE
Uni_10	85.80%	1.50 m
Uni_20	89.22%	1.24 m
Ran_10	55.49%	5.16 m
Ran_20	62.40%	4.60 m

## Data Availability

Not applicable.
